# Sex Work Criminalization Is Barking Up the Wrong Tree

**DOI:** 10.1007/s10508-017-1008-3

**Published:** 2017-06-05

**Authors:** Ine Vanwesenbeeck

**Affiliations:** 1 Rutgers, Expert Centre on Sexuality, PO Box 9022, 3506 GA Utrecht, The Netherlands; 2 Interdisciplinary Social Sciences, Utrecht University, Utrecht, The Netherlands

**Keywords:** Sex work, Trafficking, Criminalization, Human rights, Public health

## Abstract

There is a notable shift toward more repression and criminalization in sex work policies, in Europe and elsewhere. So-called neo-abolitionism reduces sex work to trafficking, with increased policing and persecution as a result. Punitive “demand reduction” strategies are progressively more popular. These developments call for a review of what we know about the effects of punishing and repressive regimes vis-à-vis sex work. From the evidence presented, sex work repression and criminalization are branded as “waterbed politics” that push and shove sex workers around with an overload of controls and regulations that in the end only make things worse. It is illustrated how criminalization and repression make it less likely that commercial sex is worker-controlled, non-abusive, and non-exploitative. Criminalization is seriously at odds with human rights and public health principles. It is concluded that sex work criminalization is barking up the wrong tree because it is fighting sex instead of crime and it is not offering any solution for the structural conditions that sex work (its ugly sides included) is rooted in. Sex work repression travels a dead-end street and holds no promises whatsoever for a better future. To fight poverty and gendered inequalities, the criminal justice system simply is not the right instrument. The reasons for the persistent stigma on sex work as well as for its present revival are considered.

## Introduction

Sex work policy regimes have always and everywhere formed a prominent chapter in the field of sexual politics. Around the world, criminalization is the dominant state policy vis-à-vis commercial sex. The philosophy behind criminalization is largely of an abolitionist nature and starts from a strong moral rejection of sex-for-pay and from the idea that casting the practice as illegal holds the best cards to, ultimately, get rid of it altogether. Either selling sex, organizing it, buying it, or all of these may be met with punitive measures under criminalization. Regimes vary in the fierceness employed in policing and enforcement of the law. Traditionally, in many countries (among which most of the U.S.), selling and organizing are actively tracked down and prosecuted.

Decriminalization, on the other hand, is still extremely rare. Decriminalization implies that no particular laws other than regular employment laws address commercial sex. It starts from an acknowledgement of sex work as work and has the explicit ambition to support the empowerment of sex workers as workers and to reduce the stigma on sex work. In New Zealand, decriminalization has been the official policy since 2003 (for natives and people with residence permits) (e.g., Abel, Fitzgerald, Healy, & Taylor, 2010).

Other countries have introduced regimes of partial legalization. Legalization is often heavily regulatory and typically sets the limits of legality through an extended set of conditions under which sex work is provided legal status. These conditions may relate to sex workers’ age and immigrant status, recruitment strategies, mandatory registration, health checks, geographical locations, building regulations, etc. Regulation or legalization is typically pragmatically motivated by law-and-order-type intentions and a (HIV and other STI’s) risk-reduction impetus. Countries characteristically associated with legalization are the Netherlands and Germany. Sex work, where permitted, is treated as work and emancipatory ambitions are explicitly formulated. However, as the case of the Netherlands shows, these ambitions are implemented half-heartedly (Vanwesenbeeck, 2011) and have so far not led to a significant improvement in sex workers’ social status. Meanwhile, regimes of limited legality, in the Netherlands as in other European countries, seem to become increasingly “rule-heavy,” to the extent that many sex work practices are actually penalized. Legalization principally does not exist without criminalization. It is all a matter of balance which one has the upper hand.

At present, a growing intention to control and punish has been observed in the Netherlands (e.g., Outshoorn, 2012) and Germany, as in other (notably European) countries (e.g., ICRSE, 2016a; Persak, 2014; Skilbrei and Holmstrom, 2013). This “regulation to deter” (Dolinsek, 2016) is part of a broader development in which sex work is (again) increasingly framed as extremely problematic and in which repression is spreading. “New” forms of criminalization are progressively more popular. “Neo-abolitionism” has been introduced as an umbrella term to describe these recent shifts (Scoular & Carline, 2014; Ward & Wylie, 2016).

### Neo-Abolitionism

Two aspects strongly characterize neo-abolitionism. First, neo-abolitionism is underpinned by a remarkable revival of anti-trafficking discourses in sex work policy and public debates. Sex work policies seem to now have been reduced to policies against trafficking. The UN Trafficking Protocol from 2000 and the North American “war-on-trafficking” initiated by the Bush regime have no doubt had an impact on Europe (and the rest of the world). European countries are now also spending vast amounts of funds on anti-trafficking initiatives (Hoff, 2014). However, there is huge (national and) international confusion over definitions of what exactly counts as trafficking (Wijers, 2015). Trafficking figures have been revealed to be inflated and ill-founded (Weitzer, 2015). The Netherlands with their elaborated system of registration are inflating trafficking figures by counting not only actual cases, but also “possible victims,” a qualification strongly subject to opinion and profiling. Fact is, the dominance of the anti-trafficking discourse maintains a view of sex work as violence and of sex workers as victims, a view that hides voluntary (migration for the purpose of) sex work from the eye and strongly fuels punitive legal practices vis-à-vis sex work.

Secondly, many countries (e.g., Canada and a growing number of countries in Europe) have now turned to criminalizing the buying of sex, also called “the Swedish model” as Sweden was the first to introduce its Sex Purchase Act in 1998 (e.g., Ekberg, 2004). Client criminalization rests on the idea that “ending demand” will ultimately abolish sex work and is therefore markedly abolitionist in nature. The “end demand” approach has been aggressively marketed by Sweden as a feminist policy par excellence (Florin, 2012; Outshoorn, 2015). As a result, the European Parliament endorsed an advisory motion promoting “demand reduction” among its member states in 2014, while in 1986 the Parliament of that time still recommended decriminalization of the “exercise of the profession” (Euchner & Knill, 2015). Although the most basic tenet of the Swedish model is the non-criminalization of sex workers themselves, many countries, including Sweden, adopt the model while definitely not abstaining from the active and ongoing harassment or even persecution of sex workers in the meantime (Levy & Jakobsson, 2014; Smith, 2016).

### This Essay

The spread of neo-abolitionism forms the backdrop to review what we know about the effects of punitive and repressive regimes vis-à-vis sex work. From the evidence presented, I will brand sex work repression and criminalization as “waterbed politics” in that they push and shove sex workers around with an overload of controls and regulations that in the end only make things worse. In my view, commercial sex is not only widely prevalent but a basically fully legitimate form of sexual relations, provided it is consensual, worker-controlled, non-abusive, and no more exploitative than other jobs would ideally be. I intend to illustrate how criminalization and repression make these conditions less likely. Moreover, they completely fail to support sex workers and victims of trafficking alike in advancing the circumstances of their lives and work. Nor do they contribute in any way to an improvement of the conditions (the ugly face of) sex work is structurally rooted in. These conclusions then lead to the basic question of why commercial sex is so fiercely condemned in the first place and, moreover, why there seems to be an apparent revival of those sentiments.

## The Harmful Workings of Criminalization

There is a steadily growing literature evincing how a repressive approach toward commercial sex is at odds with public health and human rights principles (cf. Platt & Grenfell, 2016). The direct and indirect pathways through which criminalization exerts harm are becoming increasingly understood. One of the key principles is that criminalization fuels stigma, by framing commercial sex as immoral, illicit, and unlawful, by declining sex workers’ (human and worker) rights and by powering negative opinions. Stigmatized people imputed a “spoiled identity” (Goffman, 1963) run a higher risk of being undervalued, socially excluded, and discriminated against. Some of the specificities at work in the case of sex workers are described below.

### Escalating Risks and Vulnerabilities

The risks of STI/HIV infection and of physical and sexual violence have traditionally been associated with sex work. Recently, *The Lancet* published some systematic reviews that provided compelling evidence of the harmful workings of criminalization in these contexts. Shannon et al. (2015) reviewed 87 studies on sex workers’ HIV risk. They modeled the reduction of potential HIV infections through structural changes in regions with high HIV prevalence among female sex workers and found that decriminalization of sex work would have the greatest effect of all on female sex workers’ HIV risk across all settings. It was calculated that decriminalization could avert 33–46% of HIV infections in the coming 10 years, provided it was accompanied by sex worker-led interventions and community empowerment. Shannon et al. predicted notable reduction in client violence and police harassment, safer work environments, and increased condom use as a result of decriminalization. Studies reviewed indicated that the risk of HIV/STI transmission was two to four times amplified among sex workers with criminalization-related experiences, such as the experience of prison or arrest, having sex with police officers to avoid arrest, having condoms or needles and syringes confiscated by the police, or having been subject to police raids.

Deering et al. (2014) systematically reviewed 42 international studies on sexual and physical violence against sex workers. They calculated that the risk of violence was amplified up to seven times among sex workers with criminalization-related experiences. Oppression and criminalization make sex workers vulnerable to violence from managers, clients, and other individuals, from social services, the police, immigration officials, and the judiciary (see also ICRSE, 2016b). The review indicated that street-based workers, migrant workers, drug-addicted workers, and transgender workers were particularly vulnerable. Criminalization (including oppressive anti-trafficking and migration policies) produces (sexual) abuse and exploitation of sex workers, because the whore-stigma legitimizes all sorts of presumptuous behavior and supports a culture of impunity for violence and aggression. Besides, illegality and stigma denies sex workers equal protection under the law and forecloses them taking recourse to the courts. Millions of sex workers around the world are not able to rely on the police for protection, but rather run the risk of being fined or incarcerated themselves when reporting crimes against them.

Indeed, in many countries around the world, the police are sex workers’ worst enemies. State oppression of sex work and sex workers may be accompanied by many blatant human rights violations, such as assault and harassment by police officers, naming and shaming, being outed vis-à-vis third parties (such as landlords), extortion and blackmail, arbitrary arrest and detention, inhumane conditions of detention, unlawful profiling, exploitation and bribery, confiscation of property, child custody disallowance, forced rehabilitation, expulsion and deportation, and denial of access to justice (e.g., Global Alliance Against Traffic in Women, 2007; Women’s Legal Centre, Sisonke, & The Sex Workers Education and Advocacy Taskforce, 2012). In addition, the so-called rescue industry (Agustín, 2007) may also do substantial harm to sex workers by, often aggressively, raiding and closing sites and displacing sex workers. Empower Foundation (2012) in Thailand suggested that rescuers actually posed a greater threat to the safety of sex workers than traffickers.

Trafficking may be legally defined in such a way that sex workers’ regular practices of sharing space, sharing information, and dictating the manner in which they conduct their business are considered trafficking (e.g., Burns, 2015). Anti-trafficking measures often seriously harm the people they are supposed to protect. Many regulations actually create mechanisms for abuse by authorities and others and invariably translate into discrimination and exploitation. Migrant sex workers are the most vulnerable in many respects. When migrating to a country where sex work is heavily policed, migrant women even only suspected of being involved in sex work run immediate risks of being expelled and deported or sentenced to re-education camps (Corrêa, De la Dehesa, & Parker, 2014). These are clear cases of migrant sex workers being denied the right to self-determination and basic freedom of movement. When ending up in anti-trafficking raids, migrant women are often the first to be violated. In the moral and social panic surrounding sex work and trafficking, migrant sex workers are undoubtedly hit the hardest.

Clearly, actually becoming the victim of trafficking that entails coercion, deceit, and aggression comprises a serious form of violence and human rights violation. However, the prevalence of these forms of trafficking and their relation to sex work criminalization are difficult to establish. This is exacerbated by international confusion around what exactly comprises trafficking and uneven registration practices. Criminalization may actually attract trafficking because it increases sex workers’ dependence upon profit-seeking entrepreneurs, pimps, smugglers, and all sorts of go-betweens (Vanwesenbeeck, 2011). Because of sex workers’ lack of rights and possibilities, a market is created for those who, be it through benevolence or malevolence, offer to assist them in organizing their (international) travel, their work, and their lives. Organized crime is often seen to flourish in situations of criminalization and illegality. But a review of 46 studies from various Northern European countries commissioned by the Dutch Ministry of Justice (Lensvelt-Mulders, Lugtig, Bos, Elevelt, & Helms, 2016) concluded that the studies were neither reliable or valid enough to provide dependable estimates of the effects of prostitution policies on the prevalence of human trafficking.

What is clear, however, is that criminal law relating to trafficking as a rule does not treat victims very well. When reporting trafficking and other abuses, victims may be met with demands to stop working sex and thus refrain from earning an income, they may be deported, and they may be offered insufficient witness protection. When not accused of lying and/or incriminated themselves, that is. Sex workers’ lack of recognition before the law under criminalizing policy regimes not only harms sex workers but also strongly hampers the fight against trafficking and other abuses.

### Negatively Impacting Working Routines and Relations

There is agreement in the literature that sex work repression doesn’t stop sex work but drives it into more covert forms where working routines are negatively impacted (e.g., Decker et al., 2015; Harcourt, Egger, & Donovan, 2005; Urada, Goldenberg, Shannon, & Strathdee, 2014). With increased prosecution, regulation, or registration demands, sex workers have an amplified interest in remaining out of sight of the authorities. Under regulatory regimes, an “illegal” circuit is called into being involving sex workers who choose to work outside of the system. This movement underground is also a push into further social isolation, because peer and outreach networks are likely to be disrupted. When brothel keeping is illegal, sex workers will often choose to work alone, in more dangerous isolation. When clients are criminalized, regular safety precautions become more difficult to put into practice and client interactions and working routines are more dangerous. Studies show that negotiations with and screening of clients are more hurried and less careful under client criminalization, with clients understandably less willing to reveal requested information about themselves (Dodillet & Ostergren, 2011; Levy & Jakobsson, 2014).

More difficult working routines increase economic pressure and decrease sex workers’ control over their work since they will face fewer possibilities for setting the conditions under which they provide their services. Generally speaking, sex workers’ social status and control over sexual and employment-related negotiations are reduced by stigma. Under conditions of stigma, sex workers are actively discouraged to set “too many” demands or advance their terms “too strongly.” Managers as well as clients argue that “you cannot be too choosy when you decide to do this work” (Vanwesenbeeck, 1994; see also Lister, 2015). In the Netherlands, club and brothel managers see their power increase as progressively stricter regulation and gentrification have foreclosed many possibilities to work independently and sex workers are competing for sites in which to work. Generally speaking, stigma, discrimination, and rightlessness of sex workers invariably work to the advantage of managers, smugglers, in-betweens, and (other) people without scruples. When strategies try to “save” sex workers rather than empower them, all parties profit except sex workers themselves.

A study of my own, showed that stigma also negatively affects job-related psychological stress (Vanwesenbeeck, 2005). I focused on burnout symptoms (emotional exhaustion, depersonalization, and reduced personal competence) among indoor female sex workers in the Netherlands and found that burnout was not as much related to concrete job characteristics (such as financial rewards, number of working hours, number of clients) as it was to the experience of negative social reactions to doing sex work, to role conflict, to experiences with violence, and to a lack of a worker-supportive organizational context. It was concluded that burnout as a measure of psychological stress is not so much associated with sex work per se as with the stigma associated with sex work. Likewise, Platt and Grenfell (2016) concluded, following a review of the literature, that sex workers’ emotional health is, to a large extent, shaped by stigma-related factors such as being discriminated against, difficulties combining work and home, and fear of being found out.

Last under this heading but certainly not least, it must be stressed that criminalization, by not acknowledging sex work as work, principally violates sex workers’ rights as workers. There are no routines or institutions officially representing their interests in terms of working conditions and relations. In addition, criminalization and stigma may hamper sex workers’ information-seeking, support and training, self-organizing, advocating for their rights, or reporting of mistreatment by managers or colleagues alike. Unionization is not facilitated in any way under criminalizing regimes. Illegality is a notable disempowering condition, of sex workers as individuals and as a professional group. Moreover, laws criminalizing sex work, casting of sex workers as victims rather than as workers, and the dominance of rescue and rehabilitation discourses, form important barriers to much needed implementation and scaling up of community empowerment interventions (Kerrigan et al., 2015). Limited organizing experience at the level of the community itself adds to this. Extra investments, monetary and otherwise, are needed to empower sex worker organizations to adequately serve their interests. Sex workers themselves seem to be ready. There are an estimated 250–300 sex worker organizations worldwide of which over 240 are also a member of the umbrella organization Global Network of Sex Work Projects.

### Reducing Access to Health Care

Under oppressive regimes, many direct and indirect mechanisms reduce sex workers’ access to health information, prevention, care, and services. A very direct one is when carrying condoms is seen as evidence of a criminal act and the police confiscate them to be used in courts of law (UNDP, 2012). The “push underground” also negatively affects sex workers’ visibility and approachableness by outreach and harm reduction services. Sex workers themselves show reduced willingness to access health provisions under repressive regimes. Lazarus et al. (2012) conducted a multivariable analysis with a sample of street-based workers in Canada and found that, after adjusting for sociodemographic, interpersonal, and work environment risks, occupational sex work stigma (defined as hiding involvement in sex work from friends, family or home community) remained independently associated with an elevated likelihood of experiencing barriers to health access. As Bekker et al. (2015) concluded in their review, stigma and criminalization increase the gap between sex workers and health service provision. Stigma and criminalization form barriers to effective interventions, such as condom promotion, HIV counseling and testing, STI prevention and treatment, gender-based violence prevention, and economic and community empowerment.

The quality of health services provided also deteriorates as a consequence of criminalization and stigma. Prejudice and negative attitudes among service providers upsurge, rendering their service provision far from “sex worker friendly.” A study by Scorgie et al. (2013) in four African countries entitled “We Are Despised in the Hospitals” is a good illustration. Scorgie et al. documented numerous unmet health needs, including diagnosis and treatment of sexually transmitted infections and insufficient access to condoms and lubricant. Denial of treatment for injuries following physical assault or rape and general hostility from public-sector providers was also found to be common. In the USA, a policy of willful ignorance of sex workers in HIV research and strategies is employed, resulting in ineptness, because “you cannot fix what you will not face” (Forbes, 2015). Evaluating “The Swedish Model,” Levy and Jakobsson (2014) cited a Swedish social worker as saying “I don’t spend my energy on this group of people.” According to the same study, and in line with the intentions of the Swedish “Sex Purchase Act,” social support is frequently only provided on the condition that the client leaves sex work. Thus, sex workers have to adopt a victim status to be eligible for support; otherwise, they are pushed back onto the street. After decriminalization, deeply rooted stigma and perceptions of sex workers as victims among health care workers do not vanish easily, as research in New Zealand has shown (Wahab & Abel, 2016). No matter which policy rules, many in the helping professions disproportionally meet individuals who are struggling with some aspect of their lives. Training, awareness raising, and capacity building interventions among health care professionals, the police, and the judiciary need to be high on the priority list of interventions toward improvement of sex worker health and well-being.

### Denying Self-Determination and Authority

Criminalization fundamentally violates people’s right to professional, sexual, and bodily agency and self-determination. Moreover, a choice for sex work is often discarded as not a choice at all. Those in support of sex work abolition and criminalization consider sex work to be so debased, devoid of meaningful human value, so inherently intolerable that they figure that no rational person could freely choose it for themselves. Something must be wrong with the person who makes such a choice, they are “not rational, or they are victims of coercion or deception, that is to say victims of trafficking” (Ditmore, 2008). As a consequence, states worldwide now violate sex workers’ rights with the argument that they are protecting them. In a vision of sex work as inherently violent and the sex worker as the ultimate victim, sex workers’ agency and self-determination are blatantly negated.

One of the consequences of the denial of sex worker’s rationality and their often ruthless persecution is that the relationship between sex workers and the authorities becomes notably negative and distrustful. Sex workers are not invited as discussion partners in the design of policies that deeply affect them. Nor are they valued as useful allies in the fight against abuse and crime in commercial sex. Sex worker organizations’ slogan “Nothing About Us Without Us” calls for adequate participation in policy development. Likewise, criminalization of clients pushes clients away from the police rather than invites them to come forward with the knowledge that they have about the sector. But sex workers and their clients are uniquely positioned to detect cases of exploitation and abuse (GAATW, 2007). In the Netherlands, there are positive experiences with a hotline for clients where they can report evidence of abuse and exploitation anonymously (de Groot, Haverlag, & Vogelaar, 2003). In India, there have been positive outcomes from involving sex workers in so-called self-regulatory boards working toward the removal of minors and “unwilling women” from sex work (Jana, Dey, Reza-Paul, & Steen, 2013). Wagenaar (2014) has argued for forms of collaborative governance in which government and non-government actors, in this case sex worker representatives, are collectively involved in building a legal framework that is inclusive and reflective of the reality of the divergent lives of women (and men) who engage in sex work. Such processes are bound to be far more effective than frameworks in which sex workers and clients implicate themselves when reporting cases of abuse.

### Blocking Ways Out

Although criminalization is intended to “save” sex workers from working sex, criminalization is actually blocking ways out. In countries where policing is fierce, sex workers may be fined over and over again, which only obliges them to turn some more tricks. Sex workers may actually constantly be in and out of prison, a so-called revolving door situation. This hardly stimulates progress on the difficult route toward alternative lives and livelihoods. Generally speaking, stigma is notably disempowering. Stigmatized people see their social acceptability reduced, important social and economic opportunities blocked, and their overall life chances diminished. Some of the underlying processes are self-evident: when criminalized and fined, arrested or incarcerated, sex workers are left with criminal records that may lead to difficulties obtaining legal employment, housing or government benefits. Generally, the burden of stigma affects social interaction in more or less subtle ways, through social isolation, stereotyping, generalization, attribution of negative characteristics, and enhanced aggression. The consequences thereof only make things worse for sex workers and diminish their quality of life and overall life perspectives. Criminalization often causes serious boomerang effects. Criminalization and otherwise repressive policies appear to give rise to exactly those effects they claim to fight: sex worker vulnerability and precariousness.

Sometimes designers of punitive laws do realize that sex workers suffer as a consequence of them. The Head of the Swedish Trafficking Unit, Ann Martin, has, for instance, been cited as saying, “I think of course the law has negative consequences for women in prostitution but that’s also some of the effect that we want to achieve with the law” (Costa-Kostritsky, 2014). Policy texts may pay lip-service to complement repression with social work interventions assisting sex workers in making alternative choices, but the implementation of such intentions often lags behind long after policing and repression have intensified. In Sweden, for instance, it has been shown that after implementation of the “Sex Purchase Act,” the social interventions that were also supposed to be realized were hugely insufficient as a consequence of a noted lack of capacity and legal means for their actual employment (Florin, 2012; Jordan, 2012). In the Netherlands, some stepping-out programs have been government funded, but largely to the detriment of serious attention to sex worker empowerment (Vanwesenbeeck, 2011). In addition, even in a rich and prosperous country like the Netherlands, sex workers’ professional alternatives often turn out to be so hard to reach or unattractive that the successes of stepping-out programs are remarkably thin. In the city of Deventer, only one of 25 sex workers who agreed to participate in a stepping-out program in 2011 lived up to the criteria of having found “new perspectives” after a year of program implementation (Partners & Proppen, 2011).

### Barking Up the Wrong Tree

All in all, policies that criminalize sex work are barking up the wrong tree. Laws that exceptionalize and criminalize sex work, sex workers, or their clients fail to address the structural conditions that commercial sex (its ugly sides included) is rooted in. Much repression is implemented with the conviction that making sex work as unappealing as can be will, in the end, be able to abolish it. But as long as structural conditions in terms of global economic disparities, gender injustice, poverty among women, gendered labor markets, and double sexual standards continue to exist, sex work will remain one of few moneymaking options for quite a number of women (and some men). No wonder sex work criminalization is ineffective.

Of old, the extremely high costs associated with its enforcement, its sensitivity to corruption, and its low effectiveness in terms of sex work reduction have been known (e.g., Harcourt et al., 2005; Vanwesenbeeck, 2001). “Prostitution laws don’t work because sex workers still do,” sex worker organization COYOTE already advocated in the 1980s. The many organizations that are advocating for sex work decriminalization these days (e.g., Amnesty International, 2016) endorse that there is no empirical evidence to support either a reduction of prostitution or increased safety or health of sex workers in countries adopting models aimed at complete criminalization or limited legality. Aiming at repression and, ultimately, abolishment of sex work is unrealistic and naïve. It is symbolic politics (Vance, 2011). I would like to brand them “waterbed politics” in that they simply move problems around by introducing an overload of regulations that in the end only make things worse. Sex work repression travels a dead-end street and holds no promises whatsoever for a better future.

Prostitution laws waste valuable resources that could be better used to implement laws that really improve sex workers’ rights, safety, and health. Sex worker organizations eloquently advocate for “rights, not rescue.” Scoular and Carline (2014) argued for a more productive use of the criminal law that complements rather than eclipses the wider social justice concerns in this arena. Lim (2007) argued that, to effectively combat trafficking, its root causes such as double standards, misogyny, racism and xenophobia, and, specifically, work deficits, labor market failures, and the disadvantaged position of women need to be addressed. Cruz (2013) argued that labor rights, even if desirable, do have their limitations and that it is poverty that needs to be fought, pulling the sex worker issue into the broader discussion on universal basic incomes.

These (and many other) scholars agree that, if one wants to address sex work as the poverty-driven phenomenon that it (also) is, it is poverty that needs to be fought, not sex work. Amnesty International (2016) rightly calls upon states to address discrimination and inequalities and provide a sufficient social safety net to ensure that no person has to rely on sex work due to poverty or discrimination and that everyone can leave when and if they choose. In addition, instead of allocating vast amounts of money and manpower to controlling, harassing, and putting pressure on sex workers, governments would do better putting all that energy in detection, prosecution, and sentencing of the real criminals, those that actually make victims. Coercion, exploitation, and rape are illegal and punishable in most legal systems nowadays, and these crimes are not essentially different when committed in sex work contexts. No need for specific legislation. This is especially true because most existing specific laws against pimping and trafficking are about sex, money, and travel—not about violence (Pheterson, 2016). Sex work laws exceptionalize sex work in unproductive ways because they fight sex instead of crime.

## Sex Work Stigma Revisited

The evidence in favor of sex work decriminalization and stigma reduction is huge. Yet, the stigma appears strong as ever, (neo)abolitionism flourishes, and the feminist controversy over sex work flares. Sex work morality politics are notably evidence-resistant and pre-scientific (Wagenaar & Altink, 2012). Abolitionist sex work policies are still mostly and incessantly rooted in a sex and gender morality that is heteronormative, traditionalist, and, not least for women, markedly sex negative. Abolitionists lament sex workers’ sexualization and objectification and disregard their agency and subjectivity. According to abolitionist morality, the commodification of sex is, almost by definition, unworthy and unacceptable. Abolitionists are preoccupied with the reputation of women and femininity, with the so-called horror of women being seen as sluts (cf. Young, 2015). Women engaging in commercial sex are considered either as villains or victims, in all cases as improper, unfeminine. The commercial sexual interaction is seen as a violation of human dignity and integrity. It is supposed to degrade women and to strengthen gender inequality. An extreme example is Burchill’s (1987) citation “When the sex war is won, prostitutes should be shot as collaborators for their terrible betrayal of all women.” From alternative perspectives such as mine, however, abolitionist morality is not only shockingly hostile but also notably immoral, discriminatory, irrational, and utterly naïve.

### Refuting an Immoral Morality

Considering the many serious harms sex work criminalization does to sex workers, the underpinning morality may well be branded as being essentially immoral. Millions of women (and men) worldwide are bullied and abused, blackmailed and exploited, stigmatized and discriminated against on a daily basis just because they ask money for sexual services. The abolitionist campaign is at odds with human rights as well as public health principles. In fact, human rights of sex workers are often not considered at all and sex worker discrimination is employed self-evidently. Sex workers are not the only ones to suffer. In spite of assertions to the contrary, abolitionist morality is actually adverse to the sexual rights and self-determination of all women and to gender equality alike. Abolitionist morality pushes women to live up to conventional, restrictive gender norms and ill-fated romantic ideals and punishes them for refusing to do so.

The basic idea that sex diminishes women is not only harmful to sex workers but to all women and is profoundly sexist and anti-feminist. Although male (and transsexual) sex workers suffer stigma as well (cf. Vanwesenbeeck, 2013), it is particularly the penetration of women by men, the bodily invasion of women by men, that is considered to be inherently degrading (for the women involved that is), a degradation that can only be mitigated by love or desire, not by money (Young, 2015). Consensual heterosex seems to automatically turn into abuse once money is involved. But sex workers don’t “sell themselves” or “their bodies,” they sell a service. And what’s more, some commercial exchanges may even increasingly mirror the traditional romance and the emotional intimacies found in “ordinary” relationships (cf. Sanders, 2005).

According to abolitionist morality, however, the commodification of love, desire, and sexuality is something altogether unworthy and undesirable. But let’s face it: wherever there is money, there is banking and there is commercial sex. And some sort of exchange is actually altogether quite common in many sexual interactions. Sex may be exchanged for intimacy, safety, love, partner appreciation and relational security, keeping the peace, and averting wrath and abuse. Sex researchers have documented plenty of “transactional sex,” “instrumental sex,” “tactical sex,” and “smart sex.” Exchange sex is far from exceptional in the whole spectrum of sexual relations.

The idea that our sexual world could ever be reduced to the heterosexual, love and marriage sanctioned variety of sexual relations must be dismissed as unrealistic and naïve, next to it being manifestly discriminatory of course. Abolitionist morality suffers from what Rubin (1984) has called the “MacDonaldization of sex”: there is no concept of benign sexual variation. And this is in addition to it suffering from sexual negativism, sexual essentialism, and blatant hierarchization in that one way to have sex is considered to be the only good way. The abolitionist philosophy first parades the naïve dream of romantic loving heterosex as universal, giving it excess value and significance (“the fallacy of misplaced scale”), which is then used against its commodification (Rubin, 1984).

Abolitionists contend that sex work is so degrading that it cannot possibly be a rational choice. But that is exactly what it is indeed for many women (and men) around the world for whom it is, economically speaking, simply the most favorable alternative. In many countries, a minimum wage can already be earned from the provision of 30–35 of the cheapest 10-min sexual services per month. Dismissing the decision to engage in such services as victimization and exploitation disregards the agency involved.

Moreover, in an abolitionist, radical feminist analysis, sex work is “fetishized as the locus par excellence*”* (Corrêa et al., 2014) of the capitalist exploitation of women. This obfuscates the extensive inequality, sexism, racism, and, indeed, (sexual) exploitation that characterizes gendered labor relations in many other branches. Sex work may certainly reflect gender inequality, as many other (professional and sexual) phenomena do, but it is not unequal in and of itself. Actually, in many contexts (see Seshu & Pai, 2014 with respect to India), sex workers have been shown to have more control over their bodies and their lives than other (married) women have. In addition, the decision to engage in sex work may also well be guided by an ethic of fun, sexual experimentation, and freedom (cf. Bernstein, 2007). “Good girls go to heaven, bad girls go everywhere” was a slogan once used to stress its attractive and liberating aspects.

That slogan illustrates exactly the fundamental difficulty with sex work under traditionalist morality: it being at right angles with norms of female sexual submissiveness and modesty and the societal restriction on women’s sexuality. Traditionalists reject commercial sex and other non-normative sexualities as threats against the natural order of things: the idea, based in a “domino theory of sexual peril” (Rubin, 1984), that if sexual conventions are not adhered to, chaos and anarchy will follow. The criminalization of commercial sex is a crucial instrument in the social control over women’s sexuality. Sex work has also been analyzed as being a challenge to the customary public/private dualism and erotic/economic split (e.g., Nussbaum, 1998; Zatz, 1997). The stigma is said to refer to deeply felt anxieties about women trespassing the dangerous boundaries between private and public. The criminalization of sex work is proposed to be an attempt to force back public elements of sex work into the realm of private sexuality, thus keeping the economy and sexuality symbolically separated (and leaving female unpaid labor unrecognized). After all, what if all women would start charging for sexual services!?

### Why a Revival?

These analyses do not explain why there is a prominent resurgence of sex work rejection and criminalization in this present day and age, including in Europe. There is hardly any evidence to tackle that question, only sensible suggestions. It is possible that neo-abolitionism is “just” old-fashioned morality politics exacerbated by the pendulum swinging back in reaction to the remarkable strength that sex workers’ voices and organizations have been gaining worldwide. It has also been suggested (e.g., Ward & Wylie, 2016) that increasing (social anxiety around) international migration forms a crucial backdrop against which anti-trafficking discourses now proliferate. Some scholars have indeed explicitly shown that their governments are willfully invoking emotions around “vulnerable prostitutes” and “the other” to legitimize harsh anti-trafficking legislation, to intensify the surveillance of migrating women, and to harden national security as well as international state power (see Hubbard, Matthews, & Scoular, 2007 for Sweden; Carline, 2012 for England and Wales; Pliley, 2015 and Risley, 2015 for the U.S.).

In addition, a strengthened focus on reproduction and family values seems to have taken hold, in Europe notably in the Eastern region and the Balkans. A prominent backlash against reproductive rights and an infringement on women’s rights and LGBT organizations can be observed, an overall intensified rallying against the gender and sexual equity agenda in many European countries (Kuhar & Paternotte, 2017; Kulpa & Mizielinska, 2011; Outshoorn, 2015; Verloo, 2016; Ward & Wylie, 2016). These worrying developments are bolstered by a strengthened role of organized religion and a rise of extremist right-wing parties that typically cater traditional gender ideologies and racial inequality. It may also be suggested that neo-abolitionism can be explained through its convergence with neoliberal forms of governance, within which criminalization, state securitization, and shrinkage of the welfare state are paramount. And to take it even further, a rise of authoritarianism and post-truth politics could be brought up as societal contexts in which neo-abolitionism fits quite nicely.

More concretely, the alliance between the abolitionist war on trafficking and the radical feminist anti-sexual violence movement has intensified in recent decades (Ward & Wylie, 2016). The radical feminist position that sex work is, by definition, a form of violence against women has grown into the proposition that all sex work is, by definition, a form of trafficking. This has positioned the movement as an even stronger driver of (neo)abolitionist policies than it has ever been. Radical feminist transnational networks (such as the European Women’s Lobby) have sought and established a substantial growth in power (and a substantial increase in funding) during the last decades. Their influence on state politics has augmented into forms of “state feminism,” such as in Sweden.

Of old, there have been basically two strands of feminism in relation to sexuality issues: a victim-oriented, sex negative, “radical” one and a power-oriented, sex positive, “liberal” one. The persistent controversy between the two on the issue of prostitution has been disastrous for the case of sex worker rights. My plea here is for a liberal feminism that includes sex workers, an inclusive feminism based on the understanding that women and sexual minorities, some of whom are sex workers, share the experience of living in a sexist and gender unjust society, be it from unevenly distributed positions of comfort or hardship. Those that have to navigate the least comfortable conditions should not be left behind. All forces have to be joined to put up at least some weight against the massive and expanding powers in support of gender traditionalism and economic inequality.

## Conclusion

It is time to acknowledge commercial sex as a widely prevalent and basically fully legitimate form of sexual relations. For a variety of reasons, many women (and men) will turn to making money on sex and substantial groups of people will, also for different reasons, turn to paying for it. There is nothing wrong with asking or giving money for bodily services provided it takes place under humane conditions, is fully consensual, worker-controlled, free from discrimination and violence, and no more exploitative than the average job would ideally be. Anything retracting from these qualities should be fought, but without the unproductive criminalization of the branch as a whole. After all, we don’t criminalize marriage either because there is domestic violence.

Controversies on sex work seem to get stuck in simplified, stereotypical imagery of commercial sex, an imagery that denies it being widely diverse and varied, multi-layered, and multi-determined. Simplified visions of sex work as either exploitation or choice, either violence or victory (instead of it often being both or neither), obfuscate a nuanced, complex, and adequate understanding of commercial sex and sex work realities. This complexity in sex work builds upon the complexity of the societal conditions it is rooted in the first place. There are no simple solutions when it comes to improving sex workers’ position, like there is no simple solution to fighting gender and economic inequity or violence, abuse, and exploitation. Decriminalization is an important first step, but in itself not enough. The complexity of the issues at stake calls for long-term organizing, mobilization, and community interventions and painstaking processes of raising awareness, empowerment, and building solidarity and safety nets. And progress will be partial, uneven, and never ensured. One thing is sure though: increased policing and repression of commercial sex practices are not going to help any sex worker or victim of trafficking and will only make things worse. Clearly, all crime in and beyond commercial sex needs to be fought with all the legal measures available, but to improve the circumstances of the women and men working sex or the complex gender and sexual injustices that their choices and realities are rooted in, the criminal justice system simply isn’t the right instrument.
